# From safety to efficiency: How drivers of technology adoption changed during the COVID-19 pandemic in municipal home care

**DOI:** 10.1186/s12912-025-03103-y

**Published:** 2025-04-28

**Authors:** Roger Larsson, Christofer Rydenfält, Johanna Persson, Gudbjörg Erlingsdóttir

**Affiliations:** Department of Design Sciences, Faculty of Engineering, Lund University, P.O. Box 118, Lund, SE-221 00 Sweden

**Keywords:** COVID-19, Home care, Nursing, Work environment, Digitalization, Technology acceptance, Task-technology fit, Usability

## Abstract

**Background:**

Home care underwent abrupt adaptations to handle the COVID-19 pandemic including an accelerated digitalization. While some research exists regarding the working conditions for home care personnel during the COVID-19 pandemic, few studies exist on the effect of everyday technology use in home care during the pandemic. The aim of this study was to investigate how digital technologies, introduced in Swedish municipal home care during the COVID-19 pandemic, were adopted and used by home care nurses and how well they fitted the context of use over time.

**Methods:**

An ethnographic approach was employed where qualitative data were gathered via semi-structured interviews and field observations. The data were subjected to thematic analysis. The Technology Acceptance Model (TAM) and the Task-Technology Fit (TTF) model were used as theoretical frameworks to identify and discuss factors associated with technology acceptance, use, and fit with the work.

**Results:**

Three new technologies were implemented: digital infection status lists, digital faxing, and digital meetings. Around these three technologies, two main themes related to the adoption and acceptance of technology were constructed: *Ensuring safety* and *Striving for efficiency*. Initially, the implementation of the technologies was solely driven by a need to ensure safety. However, benefits promoting efficiency were progressively discovered. After the pandemic, the perceived usefulness of the technologies was solely related to efficiency. Digital meetings continued to be in use also after the pandemic since they improved efficiency. Digital faxing continued to be in use despite being associated with usability problems, as the previous solution, the analogue fax, had been decommissioned. Thus, adoption was not only a matter of perceived usefulness but also a matter of other organizational factors and decisions.

**Conclusions:**

Technology had a central role in home care during the pandemic as it was used to ensure safety. Contextual conditions changed over time, and with them motivations to use the new technologies. The dynamic nature that dictates technology use in practice is not captured well in the TAM and TTF theoretical frameworks. A more holistic discussion is needed where context, feedback, agency, and control at work are given greater consideration and space.

**Trial registration:**

Not applicable.

## Introduction

The Coronavirus disease (COVID-19) was classified as a pandemic in March 2020 [1]. As a result, circumstances in the world changed from benign to volatile, forcing adaptations to work and life [2]. Varying strategies were implemented to hold back the spread of the virus. By 31 December 2023, nearly 774 million cases had been confirmed and 7 million people had lost their lives [3]. During the pandemic, health care and other care institutions experienced severe strain on their ability to deliver care for patients, and on their professional working conditions. Much research has been conducted on how hospital personnel performed in-patient care, such as in intensive care units [4–7]. Much less knowledge exists on the contextual circumstances for municipal care personnel during the pandemic and their working conditions.

In Sweden, responsibility for the public health care system is divided. Hospitals and community health centres are operated by regions, while municipalities are responsible for health and social care related to prevention work, elderly care, and care for other citizens in need [8]. Thus, municipal home care refers to health care provided by municipalities. Registered- and community health nurses in municipal home care (from here on referred to as home care nurses) primarily perform this service in the form of e.g. medication management and wound care, while homemaker services provide non-medical support services [9].

The Swedish strategy of maintaining a relatively open society throughout the pandemic was based on recommendations and an emphasis on individual responsibility. This had consequences for elderly care due to its inadequate measures to protect people who were elderly [10–11]. Of the over nineteen thousand who passed away with COVID-19 as the underlying cause of death in Sweden, 89% were people over the age of 70, and 69% were enrolled in municipal health and/or social care [12].

Personnel in Swedish municipal health and social care faced major challenges during the COVID-19 pandemic. While work environment related problems were common even before the pandemic [13–14], these problems intensified due to the pandemic and new problems arose. Registered nurses and managers in home care expressed increased psychosocial strain and workload, and nursing assistants and licenced practical nurses in homemaker services expressed that the pandemic brought their work to a tipping point [15–16]. Like society at large, home care underwent abrupt adaptations to handle the COVID-19 pandemic, one being a forced/accelerated digitalization process. Municipal home care has traditionally not been technologically intensive. Neither has it been fast to adopt new technologies. However, the use of welfare technologies is increasing in municipalities, especially in homemaker services and municipal home care [17]. This increase continued during the pandemic and digitalization is considered important for the development of elderly care in the future [18–19]. However, digitalization of home care is difficult [13, 20–22], and few studies exist on the effect of everyday technology use in home care. Challenges and opportunities in home care nursing practice identified prior to the pandemic were concerned with information access and documentation in the field, communication, and increased complexity due to digitalization [23]. Home care nurses have also been found to perform networking practices around their patients by which communication through a multitude of media affected their work environment [24–25]. This indicates that digitalization has played and can play an even more important role in home care.

The aim of this study was to investigate how the digital technologies that were introduced in Swedish municipal home care during the Covid-19 pandemic were adopted and used by home care nurses, and how well they fit the context in which they were used. More specifically, by following the changing roles of technology over time during the pandemic, the research presented explores how the role of technology in practice changed over time and the effects that the technology had on the nurses’ work environment. To interpret and discuss the findings, the theoretical frameworks of the Technology Acceptance Model (TAM) and the Task-Technology Fit (TTF) model were used post data collection to discuss the adoption of new technologies and how well they fit with the nurses’ tasks at different times [26–28].

## Theoretical framework

The following two sections describe the TAM and TTF theoretical frameworks. These frameworks were chosen to investigate how the role of technologies changed over time and their effects on work. Several theoretical models and perspectives of technology acceptance and change exist with varying complexity. TAM is one of the most influential theoretical frameworks in the field of technology acceptance. In this research, TAM is used to explore the motivational factors behind the use of technology. TTF, on the other hand, is used to describe the impact that technology has on work over time due to the characteristics of the tasks, the technology, and their fit. A combination of these theoretical frameworks, the TTF-TAM, exist [29]. However, here, TAM and TTF were chosen over more comprehensive models due to their simpler design. As theoretical models grow in scope, they also tend to become more complex. This, in turn, can make them more difficult to apply to qualitative data.

### Technology acceptance model

**Fig. 1:**
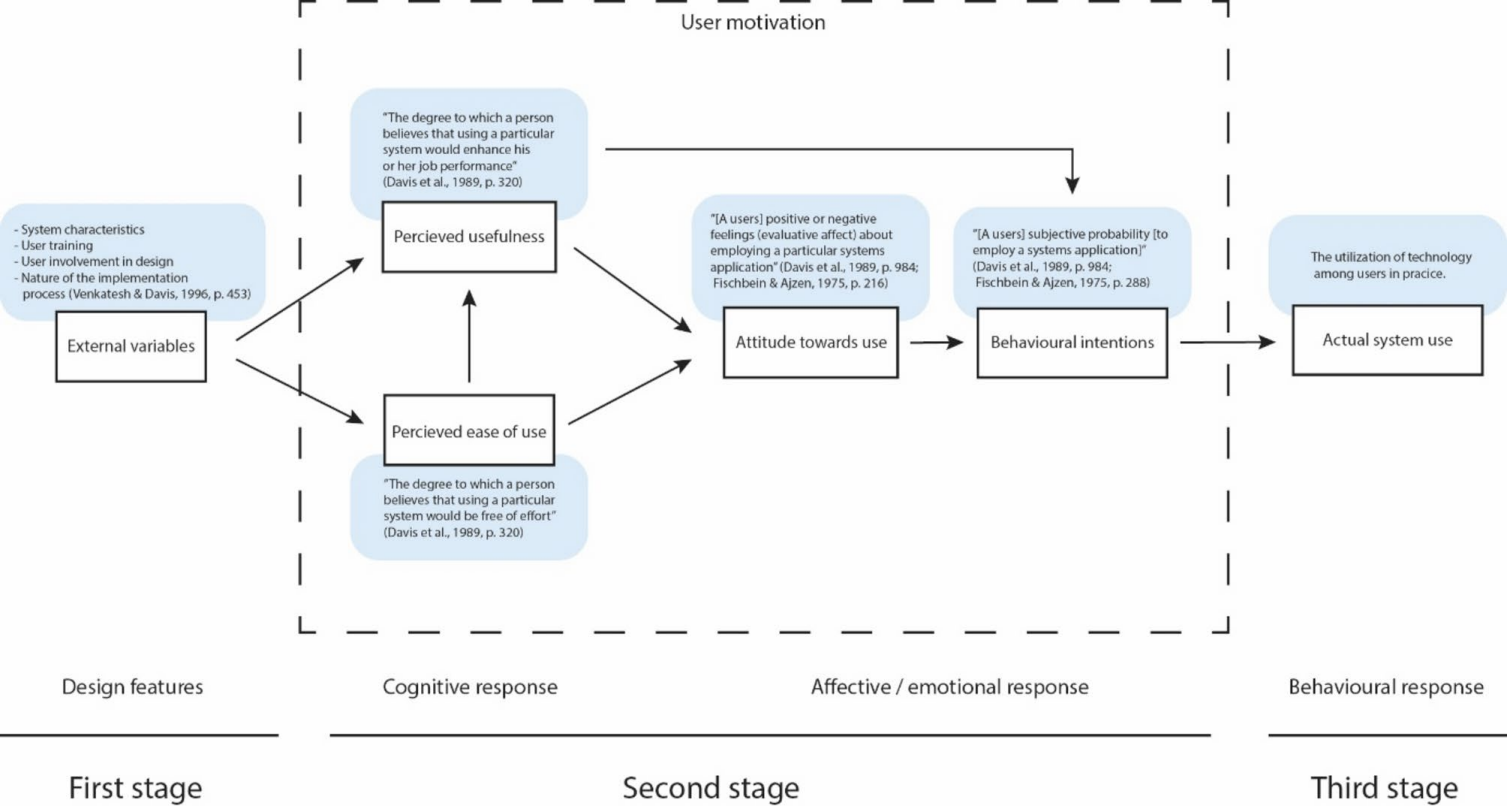
The Technology Acceptance Model (TAM) after [28] comprised of three stages and six factors

The Technology Acceptance Model (TAM) was originally introduced by Davis [30] to identify factors causing potential users’ acceptance and rejection of information technologies. TAM is based on the Theory of Reasoned Action (TRA) and has become one of the most influential technology acceptance theories. TAM has been extended and modified several times, this includes e.g. TAM2, TAM3, and UTAUT [31–34]. The classic TAM is illustrated in Fig. 1.

TAM postulates that technology acceptance is a stage process comprised of six factors.

The first stage – design features – consists of “*external variables*”, such as the technology’s characteristics, its implementation process, and user training and design involvement [31, p. 453].

These variables trigger the second stage – the users’ cognitive and affective/emotional responses – which is comprised of four internal factors. The cognitive response includes “*perceived usefulness*” and “*perceived ease of use*”. “Perceived usefulness” is defined as “the degree to which a person believes that using a particular system would enhance his or her job performance”, with the word “useful” defined as “capable of being used advantageously” [26, p. 320]. “Perceived ease of use” refers to “the degree to which a person believes that using a particular system would be free of effort”, where “ease” is defined as “freedom from difficulty or great effort” [26, p. 320].

An affective/emotional response is then triggered, influenced by the user’s cognitive response, comprised of the two factors “*attitude towards use*” and “*behavioural intentions*” to use technology. “Attitude towards use” can be defined from the related “attitude towards behaviour” in TRA as “[a users] positive or negative feelings (evaluative affect) about [employing a particular systems application]” [28, p. 984; 35, p. 216]. In the same manner, “behavioural intentions” can be defined as “[a users] subjective probability [to employ a systems application]” [28, p. 984; 35, p. 288].

This leads to the third stage – behavioural response – manifested as the “*actual system use*” factor.

The two factors in the second stage: “perceived usefulness” and “perceived ease of use”, are the strongest predictors for technology acceptance in TAM. This theoretical framework aims to trace how individuals’ internal beliefs, attitudes, and intentions are affected by external factors.

In the field of health informatics, TAM is frequently used in studies concerned with telemedicine, electronic health records, and mobile applications [36]. The model’s application often involves the integration of additional components from other theoretical models intended to improve its predictions. This has produced contextualized versions of TAM for different care settings. Many of the studies concerned with technology acceptance in home care are concerned with patients rather than professionals. Studies on home care professionals have applied both the original TAM and extended models that incorporate factors such as e.g. subjective norms, self-efficacy, habits and job relevance [e.g. 37–38].

### Task-technology fit model

**Fig. 2:**
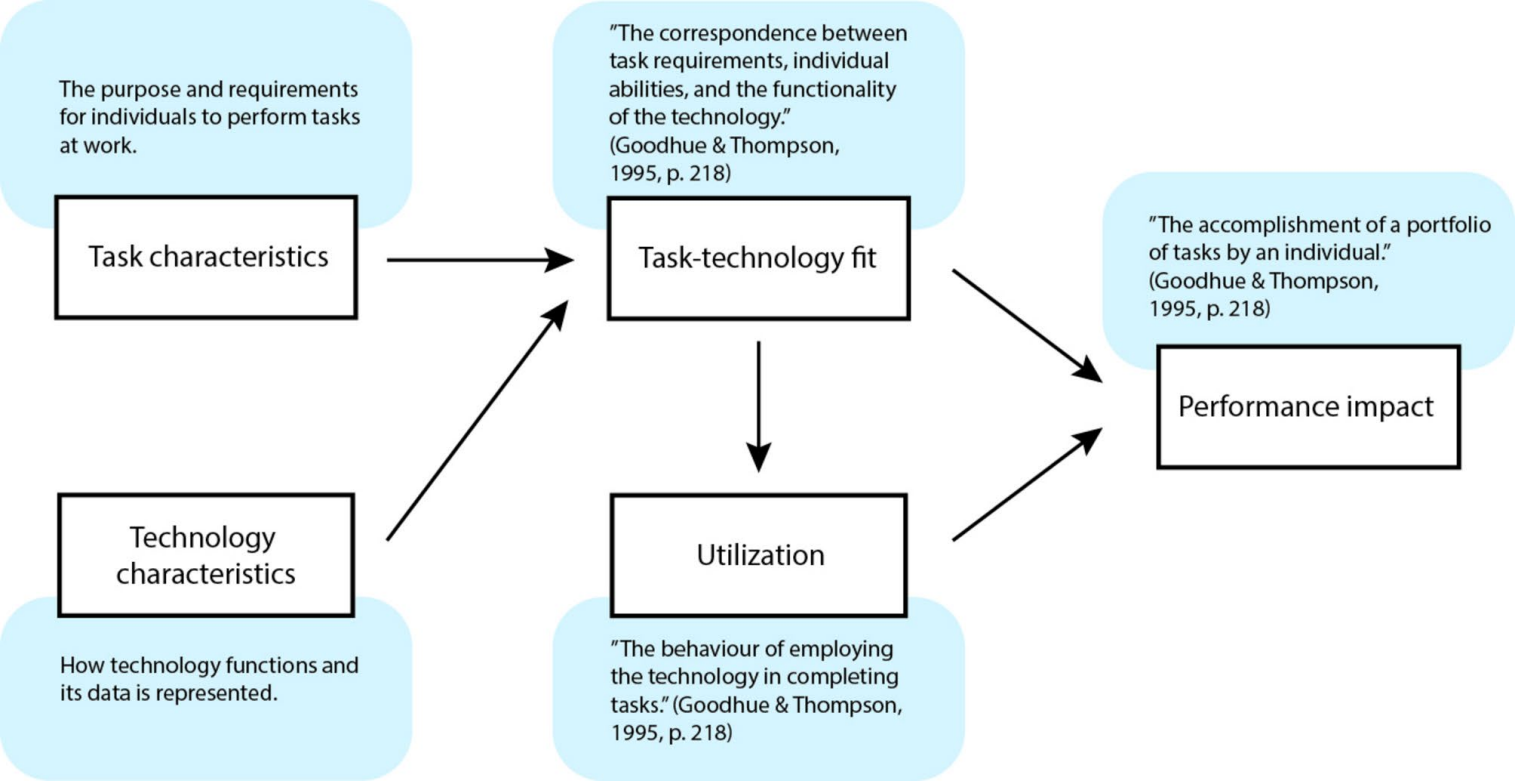
The Task-Technology Fit (TTF) Model after [27]

The Task-Technology Fit (TTF) Model was introduced by Goodhue and Thompson [27]. It examines the fit between technology and user tasks/requirements to explain how it leads to performance impact. The model is illustrated in Fig. 2.

“*Task characteristics*” and “*technology characteristics*”, respectively, influence their mutual fit, which determines how well the technology assists the users in performing their tasks. The factor “*task- technology fit*” is defined as, “the correspondence between task requirements, individual abilities, and the functionality of the technology” [27, p. 218]. This fit in turn influences the “*utilization*” of the technology, or “the behaviour of employing the technology in completing tasks” [27, p. 218]. The “*performance impact*” is the combined result of “task-technology fit” and “utilization” and indicates how well the users complete their set of tasks. This theoretical framework aims to explore the fit between tasks and technology, as well as technology utilization from a post-adoption perspective.

### Methodology

#### Research design

This study was qualitative and ethnographic in its approach. It explores how the technology that was adopted and used among nurses in municipal home care changed during the COVID-19 pandemic, as well as how it fit with their work. The data set was comprised of semi-structured interviews and field observations in the form of shadowing [39–40]. These semi-structured interviews gave insight into the nurses’ personal experiences and motivations to adopt and use technology. The field observations gave accounts of how the use took place in practice. Four municipalities of varying size were included in this study. Two of the municipalities had populations less than twenty thousand each. The other two municipalities had populations ranging between thirty and fifty thousand. Data collection was part of two research projects. The first investigated work environment related effects of an interorganizational team implementation, and the second, work environment effects of the COVID-19 pandemic.

### Sample and data collection

Data were collected in two intervals during the COVID-19 pandemic: (1) Spring (May to June) of 2021, and (2) Spring (April to June) of 2022. Access to the field was strictly prohibited for an extended period due to the pandemic. To handle the situation, interviews were performed over the phone. Shadowing was put on hold until the spring of 2022.

Twenty-four home care nurses (1 man, 23 women) were interviewed in total: 14 (1 man, 13 women) in the spring of 2021 and 10 (all women) in the spring of 2022. The participants were recruited with the help of each home care organization’s unit manager. The inclusion criteria were that the participants had to be registered nurses working in municipal home care. The interviews were conducted on the telephone and were audio recorded. The interviews lasted between 28 min and 1 h 7 min (total = 17 h 16 min, mean = 43 min). The second, third and fourth authors performed the interviews. A predefined interview guide was used. The interview guide included the topics: *mobility at work*, *teamwork*, *change in work*, *work environment related challenges associated with COVID-19*, *use of digital tools and eHealth systems*, *crisis management*, and *learnings from the pandemic*. Some questions addressed technology adoption directly. Technology adoption could also be addressed indirectly as part of the conversation regarding other questions, such as work changes and communication. For example:

- *Has the way you communicate and interact with others changed during the COVID-19 pandemic?*
- *Have needs for technology changed during the COVID-19 pandemic?*
- *Are there any COVID-19 related changes that you would like to continue with even after the pandemic?*

The field observations were performed in the spring of 2022 to observe the nurses work in practice. The participants were recruited with the help of the home care organizations’ unit managers. In total eight home care nurses (2 men, 6 women) were shadowed during their entire work shifts. The observations lasted between 6 h 53 min and 9 h 20 min (total = 67 h 37 min, mean = 8 h 27 min). Observations were conducted in three municipalities, the fourth declined to participate.

The first author performed all field observations. Handwritten field notes were taken and ethnographic drawings were made in a notebook [41–42]. The observations were guided by a list of topics that targeted the same overall subjects as the interviews. The field notes and sketches were structured chronologically as events unfolded, and ethical considerations were continuously made on what and how to document.

### Data analysis

**Fig. 3:**
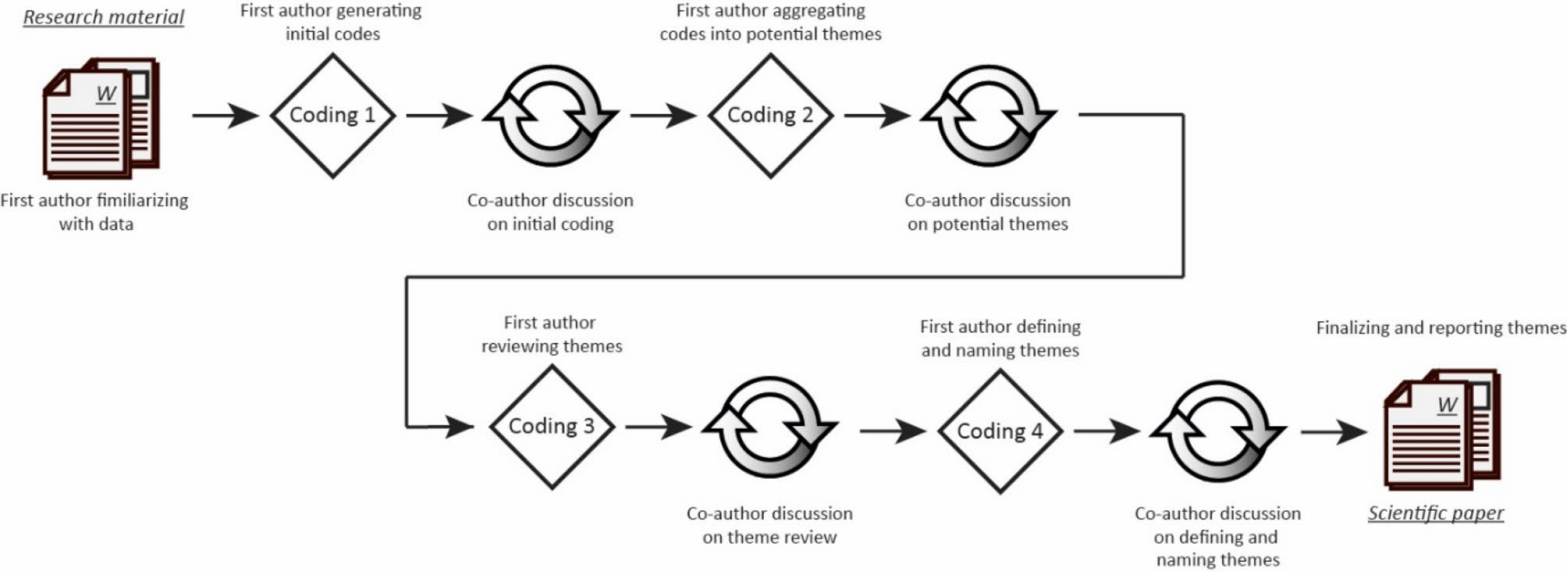
The thematic analysis process from research material to finalized analysis

All interviews were transcribed from the audio recordings. The observation fieldnotes and ethnographic sketches were converted into digital form by the first author. The interviews from 2021 have previously been independently analysed with a focus on the work environment and crisis management in home care during the COVID-19 pandemic [15]. For this study, the data were thematically analysed, inspired by Braun and Clarke [43]. The analytic process is visualized in Fig. 3.

The data were first read and listened to extensively by the first author, who afterwards openly coded the data, which resulted in a set of initial codes. The initial coding produced the following seven potential themes: (1) *As before the COVID-19 pandemic*, (2) *Effects on work and the work environment*, (3) *Information*, (4) *Knowledge*, (5) *Communication*, (6) *Changed use of technology*, and (7) *Attitudes towards using digital tools*. The potential themes were discussed in the research group. The two overarching themes addressing *Changed use of technology* and *Effects on work and the work environment* were chosen for further investigation. Together, they concerned how the use of technology among the home care nurses changed over time and how this affected their work. The data set was then reviewed and recoded from the perspective of these two overarching themes. This generated new overarching themes which were discussed among the authors until agreement was reached. The end result of the analysis process was two main themes, *Ensuring safety* and *Striving for efficiency*, which are presented in the Result section.

## Results

The analysis resulted in an insight of the role technology had for nurses in home care during the COVID-19 pandemic. This section first presents the new digital technologies introduced during the pandemic: i*nfection status lists*,* digital faxing*, and *digital meetings*. This is followed by the two themes that describe the intentions behind the technologies that were used: *Ensuring safety* and *Striving for efficiency*.

### New digital technology introduced during the COVID-19 pandemic

The utilization of digital technologies, preexisting and new, was essential for municipal home care organizations’ ability to provide care during the COVID-19 pandemic. Technology allowed the home care nurses to adapt their work as the conditions changed.

In the Spring of 2021, the work situation was highly strained due to the challenges the COVID-19 virus presented. The nurses adapted the work to be done ad hoc in order to address the evolving needs among them. Physical meetings were largely abandoned, except in isolated work groups, due to the need for social distancing. Efforts to try to control the spread of COVID-19 were important since the majority of home care patients are elderly. Still, the need for interaction and coordination among and between professional groups persisted. For that reason, interactions became increasingly technologically mediated. Practices that already existed prior to the pandemic in the municipal organizations, such as phone calls, e-mailing, and faxing, became more utilized than before. They filled the gaps created by the need for information exchange and communication. In addition, the use of three new technological tools emerged that had significant effects on the home care nurses’ work situation: infection status lists, digital faxing, and digital meetings.

Infection status lists were created by the nurses to share information about patients in relation to COVID-19. The lists were available online and managed separately outside of the municipalities’ electronic health record (EHR) systems. The lists could contain information on patient identity, COVID-19 infection, tests taken and at what time, confirmed cases of the COVID-19 infection, and the establishments of isolation/quarantine/barrier care. Table 1 illustrates the change in the use of infection status lists over time.

**Table 1:** Summary of how the use and role of infection status lists changed over time in relation to TAM and TTF

Infection status lists – TAM	Spring of 2021	Spring of 2022
External variables	*- Directives and restrictions due to Covid-19.* *- Social distancing.*	*- Directives and routines as before Covid-19.* *- No social distancing.*
Perceived usefulness	*- Gives situational awareness by providing overview over infected patients.* *- Basis for communication and information sharing about the situation among patients.*	*- No need to daily list*,* overview*,* and communicate the COVID-19 related situation among patients.*
Perceived ease of use	*- N/A*	*- N/A*
Actual system use	*- Daily use. * *- Continuously kept up to date.*	*- Abandoned.*
**Infection status lists – TTF**	***Spring of 2021***	***Spring of 2022***
Task characteristics	*- Oversee patient infections.* *- Manage testing.* *- Maintain quarantine/barrier care.*	- *Abandoned.*
Technology characteristics	*- Online and sharable document containing patient data outside EHRs.*	*- Online and sharable documents containing patient data outside EHRs.*
Task-technology fit	*- Lists used to identify needs for barrier care.*	*- Use no longer required or defendable.*
Utilization	*- Daily use.* *- Continuous information updates.* *- Designed by nurses based on perceived needs.* *- Shared across municipal instances.*	*- None.*
Performance impact	*- Situational awareness obtained.* *- Communication and information sharing essential for patient safety and occupational health.*	*- None.*

Digital faxing, also called “*e-Faxing*”, was a digital variant of the physical fax machine. Digital faxing involved sending and receiving faxes (scanned documents) electronically through the nurses’ e-mail clients on their computers instead of through physical fax machines. Two of the municipalities in this study replaced their fax machines with this practice during the pandemic, while the other two kept their faxing practice with physical fax machines. Table 2 presents the use of digital faxing over time.

**Table 2:** Summary of how the use and role of digital faxing changed over time in relation to TAM and TTF

Digital faxing – TAM	Spring of 2021	Spring of 2022
External variables	*- Directives and restrictions due to Covid-19.* *- Social distancing.*	*- Directives and routines as before Covid-19.* *- No social distancing.*
Perceived usefulness	*- Communication is freed.* *- Alleviates workload increase.* *- Maintains continuity of communication with regional health care during sick leave.*	*- Enables communication with regional health care demanded to be conducted via fax.*
Perceived ease of use	*- Continued delays in communication with regional health care.* *- Cumbersome and time consuming to use as it involves transfers between physical and digital domains.*	*- Continued delays in communication with regional health care.* *- Cumbersome and time consuming to use as it involves transfers between physical and digital domains.*
Actual system use	*- Daily use where implemented.*	*- Daily use where implemented.*
**Digital faxing – TTF**	***Spring of 2021***	***Spring of 2022***
Task characteristics	*- Communicate with hospitals*,* community health centres*,* and pharmacies.*	*- Communicate with hospitals*,* community health centres*,* and pharmacies.*
Technology characteristics	*- Electronic messaging through e-mail clients on computers.*	*- Electronic messaging through e-mail clients on computers.*
Task-technology fit	*- Digital fax used to maintain communication with regional health care also when in quarantine.*	*- Technology used to maintain pre-pandemic faxing practice.*
Utilization	*- Daily use if implemented both at the office and at home.*	*- Daily use if implemented at the office.*
Performance impact	*- Alleviates workload for healthy colleagues at the office.* *- Maintains continuity in communication with regional health care.* *- Cumbersome and time consuming to use as it involves transfers between physical and digital domains.* *- Maintained “faxing” practice.*	*- Communication bound to the office.* *- Cumbersome and time consuming to use as it involves transfers between physical and digital domains.* *- Maintained “faxing” practice.*

Digital meetings were considered necessary for social interaction. By using software that allowed video conferencing, the nurses could continue to meet among themselves and with other professional groups. Table 3 summarizes how use and the role of digital meetings evolved over time.

**Table 3:** Summary of how the use and role of digital meetings changed over time in relation to TAM and TTF

Digital meetings – TAM	Spring of 2021	Spring of 2022
External variables	*- Directives and restrictions due to Covid-19.* *- Social distancing.*	*- Directives and routines as before Covid-19.* *- No social distancing.*
Perceived usefulness	*- Enables social interaction despite social distancing.* *- Makes work more efficient as it triggers shorter meetings*,* formalized communication*,* and time savings due to less travels.*	*- Makes work more efficient as it triggers shorter meetings*,* formalized communication*,* and time savings due to less travels.*
Perceived ease of use	*- Convenient for certain meetings.*	*- Convenient for certain meetings.*
Actual system use	*- Daily to periodic use.*	*- Periodic use when convenient.*
**Digital meetings – TTF**	***Spring of 2021***	***Spring of 2022***
Task characteristics	*- Meetings in larger groups.*	*- Meetings when attendance not possible in person.*
Technology characteristics	*- Video conferencing software allowing meeting attendance online.*	*- Video conferencing software allowing meeting attendance online.*
Task-technology fit	*- Digital meetings enable social interaction and meetings despite social distancing.*	*- Digital meetings enable flexible attendance and presence at meetings when necessary.*
Utilization	*- Daily or periodic use.*	*- Periodic use when convenient.*
Performance impact	*- Necessary for social interaction and processes despite social distancing.* *- Makes work more efficient as it triggers shorter meetings*,* formalized communication*,* and time savings due to less travels.*	*- Convenient for certain meetings.* *- Makes work more efficient as it triggers shorter meetings*,* formalized communication*,* and time savings due to less travels.* *- Increased possibilities to attend education and seminars.*

Related to these three technologies, two themes emerged from the thematic analysis concerned with the intentions or drivers of use: *Ensuring safety* and *Striving for efficiency*.

### Ensuring safety theme

The most significant intent of use of the new technology in 2021 was related to safety. Safety was the highest priority behind adaptations to work during the pandemic, in line with the emphasized need to protect patients and employees from infection. It was thus the prime driver behind the adaption of new technology. Since the start of the pandemic in 2020, the work situation among the home care nurses had become highly unpredictable. This gave rise to questions on how work had to change to adapt to the new situation, including social distancing. The existing work routines and media used among nurses were adapted to fit the ever-changing needs to ensure a safety level that was as high as possible for patients and professionals alike. Measures to ensure safety were related to:

- the accessibility of and processing of information,
- the overview of infected patients,
- communications within and across organizations,
- and needs related to coordination and social interaction.

The need for an overview became imperative for the nurses during the pandemic, both for the infected patients, and for information regarding adjustments of guidelines, work routines, and their meaning in practice. Here, infection status lists provided an overview of infections among patients and made it easy to keep the information updated and to share it with others (Table 1). As one nurse put it:

“***E****very morning [we have] an assessment [about] which [patients] are isolated*,* which samples we take*,* which [patients] are negative and which are positive. And then this is sent out to the municipality*,* to managers and organizations as an infection status list. And this is done every day and every weekend. You can say that … part of the [morning] meeting is to go through the infection situation.… Then we enter another such list online when [cases] appear during the day. It is only once a day that we [go through] the infection status and [whose list is] sent out. [We have] another list in the computer where we enter [information during the day]. For example*,* if they call about a patient [who tested positive during the day]*,* I can’t wait until the next morning to let them know. Then I have to put it on the other list*,* because … what if my colleagues are going to visit that patient? They won’t see that information on the morning list. Then they have to look at the other list … which is constantly updated in terms of who is isolated*,* who is tested … So*,* it’s a super up-to-date list.” ****Home care nurse**** (Spring 2021)*.

Faxing has been indispensable for the municipal home care nurses’ communication with community health centres, hospitals, and pharmacies, since long before the pandemic. This practice had up until the pandemic been bound to the office. As the pandemic struck, this physical restriction became problematic due to the increased sick leave or care of sick children among the nurses. To maintain communication when working from home thus became crucial to cope with the work situation (Table 2). A nurse elaborates on the topic:

“***T****he downside of faxing (with physical faxing machines) if you need to fax something*,* is that you have to make sure that a nurse is at the office [to receive it]. [Even though this] was usually the case*,* [you must] keep track of the faxes that come in too. So*,* it was a bit like that [in the beginning of the pandemic] … But now we’ve got a better solution as far as faxing goes. [Now] we send faxes via e-mail [instead] as of two weeks ago.”**** Home care nurse****(Spring 2021)*.

As a result, when digital faxing was employed, it was a measure to overcome the limitations that physical fax machines had. By enabling this communication to become boundless, even from home, this provided better prerequisites for the continuity in communication. This helped continue the provision of home care and alleviate the additional job strain for the healthy nurses working at the office (Table 2). However, usability problems existed with using digital faxing as the following nurse describes:

“*[****Y****ou type the fax on the computer] … Then you have to [print] it*,* go get it [from the printer]. Then you have to scan it in [with the scanner and send it to yourself]*,* go back to the desk. Then you [download and] sign the scanned message … save it on the [computer’s] desktop*,* go into Outlook… and [only then] send it to the clinic. It’s the latest… So*,* it takes a lot of time. [Instead of just writing an email and sending it]… which we don’t get. But it would have been the best. My God… It’s cumbersome. It’s a waste of time.”**** Home care nurse**** (Spring 2021)*.

The introduction of digital meetings enabled the necessary social distancing, and upheld the necessary meeting structures at work. They primarily satisfied the needs among the nurses to maintain practices of coordination, and to achieve organizational meaning and social interaction at work (Table 3). As one nurse express:

“*[****V****ideo calls] have meant … that we’ve been able to continue to have some workplace meetings and things like that*,* that we otherwise wouldn’t have been able to do because we’re too big of a group.”**** Home care nurse**** (Spring 2021)*.

Information regarding guidelines from authorities and changes in work routines from management changed continuously as the pandemic situation evolved. These kinds of information were often distributed by e-mail and had the effect that the nurses had to continuously consider, interpret, and (re)implement new routines. Digital meetings became important here as they allowed the nurses to reach consensus on the current situation and discuss the meaning of new information (Table 3). The following nurse describes:

“*[****W****]e then started up meetings digitally*,* precisely to address the situation and be able to ask questions*,* and so on. And there was a bit of instruction and supervision*,* because you had thoughts and reflections like “How should we think there?” And then … Things change during the course of the pandemic as well. It was a little too much with all the symptoms there were. Then all of a sudden you could infect before you had any of the symptoms. Then it was probably airborne. It was such information that came through the media*,* a lot. And it’s good if you’re able to vent your thoughts. “[H]ow are we thinking here?” and “[W]hat are we going to do here?”**** Home care nurse**** (Spring 2021)*.

### Striving for efficiency theme

Already during 2021, the nurses discovered additional benefits with the use of the new technologies. These benefits accumulated into the intention of use associated with *efficiency* and worked simultaneously with *safety*, which was the dominant intention of use at the time. As the restrictions disappeared by the Spring of 2022, safety as the motivation behind use disappeared with them. Social distancing was no longer necessary due to the reclassification of COVID-19 to a non-dangerous decease. Physical meetings quickly returned as the norm for social interactions. Work had thus started to return to a “new” normal, and technology use was no longer motivated by pandemic-related safety needs. The intention of the use of efficiency, however, remained, although, the usage of the three new technologies introduced during the pandemic changed as a result of this shift.

The nurses’ use of the infection status lists was quickly abandoned when the restrictions disappeared (Table 1). Digital faxing continued to be used where it had been implemented (Table 2). And despite the return of the physical meetings as the norm, digital meetings remained to be used when needed (Table 3). While the extent to which digital meetings were used after the pandemic clearly decreased compared to during the pandemic. Instead of being part of the daily routine, digital meetings were used when requirements demanded it or when the nurses found them convenient.

*“****N****ot always*,* but sometimes we need to meet in person for certain kinds of meetings*,* and that can be nice too. But it has still been a positive thing that has come out of using more digital tools and such. And you can do it in the same way with other agencies that we work with; maybe not regularly*,* but sometimes we have been able to link up in care planning*,* or something similar*,* to make it easier to be able to participate.”**** Home care nurse**** (Spring 2022)*.

The use of digital meetings after the pandemic showed a shift in the culture among nurses related to technology mediated interaction. This was exemplified in an interview with a home care nurse:

*“****I****t seems like a new culture has arisen with [the digital meetings] and it is due to the pandemic*,* because we didn’t have that at all before … [We have no digital meetings] with patients if we are allowed to manage it ourselves*,* but it would be the nurse practitioner-meetings and other meetings of that type. It can also be with social workers*,* and so on; thinking about teams and such too. [It] feels [like it will live on] right now. It certainly hasn’t died out; there’s no reason.”**** Home care nurse**** (Spring 2022)*.

A major benefit with digital meetings in relation to efficiency was time saving (Table 3). Two nurses deliberate on this topic:

[**A**]ll our meetings were cancelled, so then… you gained some time there. And then we started up coordinating digitally, and it has worked better than expected, I think. You save a little time when you don’t need to drive away to go to a meeting, instead you just connect online.” **Home care nurse** (Spring 2021).

*“[****W****]e have actually continued to do it; it is significantly more time-saving … We usually have team meetings with the homemaker service personnel… [W]e save time [by not needing to] go there [physically], sit down and wait for everyone [to show up]… [W]hen the meeting has started*,* it starts*,* and then our time starts at that meeting… [So] absolutely*,* it saves time.” ****Home care nurse ****(Spring 2022)*.

Increased flexibility was another major benefit of digital meetings, since the nurses no longer were required to be present at a physical location for meetings (Table 3). Nurses found that being able to attend educational sessions and seminars in a resource effective manner was highly desirable because it gave them greater access to new knowledge and opportunities for learning. Fully digital or hybrid meetings increased the nurses’ possibility to participate in meetings even if unforeseen obstacles occurred.

*“****A****nd all these digital courses that you have access to. So*,* this will … continue significantly more so … The opportunity for digital training has increased. More and more courses have become digitalized compared to what there was before … So that more people can participate. Otherwise*,* it was that … you couldn’t get away*,* and it was too expensive*,* and then there was no money*,* and so on. But now there is a much greater opportunity to participate.” – ****Home care nurse ****(Spring 2021)*.

*“****W****e haven’t had much digital meetings before*,* we had big [physical] meetings and things like that… [B]ut now we have had [digital meetings]*,* and it has worked better than I expected at least… [It] didn’t feel weird*,* it was the same exchange of information as before but [without the need of going anywhere]… [For example]*,* for some nurse meetings we would meet in the municipal hall and then everyone had to get into their cars and drive there… [S]o you save some time by staying at the office [instead]…* [*Now we have switched back to the old form of meeting again]*,* although we have got this [new] little thing that you get a link*,* so if you can’t come to the meeting*,* you can connect*,* and that’s great if you [are delayed] and not finished [with your primary care tasks or have to do something urgent]… [Then] I can sit at my computer but still take part in the meeting and listen to what is being said and add something without having to drive from my workplace.”**** Home care nurse**** (Spring 2022)*.

However, opinions were split among nurses about the introduction of digital meetings. Some nurses were positive about it, others were not. They were also divided on how it would affect their work situation. Digital meetings, despite their benefits, are not complete substitutes for the physical meetings in terms of human interaction. The nurses indicated that one does not acquire the same exchange from digital meeting discussions as before, and that relational bonds were not maintained in the same manner as before. Two nurses described it like this:

*“****A****ll meetings*,* in every way*,* have become digital*,* and I can think that it’s a little … It’s not the same exchange of discussion*,* if I say so. It is much*,* much trickier to get everyone to speak*,* when you sit in digital meetings …”**** Home care nurse**** (Spring 2021)*.

*“****W****ith homemaker services*,* I think it has become – it’s this with [digital meetings] that the meetings [become better] but the personal relationship – you don’t meet them in the same way before [the] meetings [start] and say hello. Rather I think it’s gotten worse; there’s less contact*,* less team spirit … Rather*,* things have gotten worse with homemaker services because you don’t meet them… [We have more efficient meetings but a worse relationship because we don’t see each other]. That’s what I think. If I delegate people and don’t meet them*,* I don’t really know what they’re up to.”**** Home care nurse**** (Spring 2022)*.

Throughout the pandemic, online digital meetings were never used with patients or during collective home visits together with other professional groups. There seemed to exist a broad consensus among the nurses that digital meetings were not to be used with patients or during home visits if possible. Of special importance was to have general practitioners from community health centres continue to conduct home visits during the pandemic. One nurse described how they actively worked against having digital meetings with general practitioners from community health centres. In so doing, they deliberately forced the general practitioners to go out on home visits:

*“****W****e have many SIPs [Coordinated Individual Care Plans]. An SIP*,* that you do*,* should actually be followed up once a year. And now we had a lot of patients for whom we needed to draw a new SIP and follow up on the existing ones; and then [the community health centers] wanted us to [have meetings digitally] because the general practitioners preferred not to make home visits … [But] we still got the doctors out … we have said that we prefer a physical home visit. [This is] because it’s not that often that they see their patients. So if you can get a physical home visit*,* that’s actually good.”**** Home care nurse**** (Spring 2022)*.

## Discussion

As Amankwah-Amoah et al. [2] describe it, COVID-19 was an accelerator of digitalization. The accelerated digitalization of the home care nursing practice can be explained in relation to the risk levels induced by the pandemic. When the pandemic started in 2020, it resulted in a state of emergency [44–45]. The safety of both patients and home care personnel was threatened. From the perspective of TTF, adaptations to technology use during the early stages of the pandemic were driven by a desire to keep work and performance as close as possible to “the old normal” [27]. Task characteristics were preserved, when possible, in order not to increase the nurses’ workload (see Tables 2 and 3). This was because the work situation was highly strained [15]. The use of preexisting technology, such as phone calls and e-mail, was extended where it could meet vacant needs in relation to adjusted work tasks brought about by the pandemic. Nevertheless, demands for safety and social distancing required a change in tasks. The three new technologies described in the results – infection status lists, digital faxing, and digital meetings – were introduced to meet novel needs that could not be met by the preexisting technology.

From the perspective of TAM, initially “perceived usefulness”, “attitudes towards use”, “behavioural intentions”, and actual use of technology were mostly concerned with assisting the nurses to work safely [26, 28]. However, as the new technologies were used, additional benefits were discovered. This in turn created new intentions and expectations on their future use, which would coexist with the original intention for safety until the environment reverted to the more benign “new normal” in 2022. The continued use of the new technologies would be motivated now by how well their perceived usefulness matched the home care nurses’ “new” work situation. This suggests that the influences of contextual feedback and experience are important aspects of technology acceptance. That means that without the experience from the pandemic, the benefits of the technologies under investigation that were associated with efficiency might not have been discovered, and the technology thus not perceived as useful.

As the results show, the three technologies that were implemented met three different fates after the pandemic. The sudden abandonment of the infection status lists could partly be explained by their highly specialized function: to provide an overview of COVID-19 infections among patients. In other words, the list simply had no place in the nurses’ practice after the pandemic. However, their abandonment can also be explained by the fact that the nurses under normal circumstances are not formally allowed to share information on patients, not even with each other, due to secrecy [46]. The very existence of the infection status lists should thus be viewed as a testimony of how dire the needs in home care were during the pandemic. This made it necessary for the nurses to create a tool to safeguard patient safety, even though it might be unauthorized. Thus, the lists were defendable only for as long as the crisis remained.

Like the infection status lists, secrecy is a factor that also likely explains some phenomena observed in the nurses’ daily use of digital faxing. However, despite its benefits in terms of technology characteristics, nurses broadly found its use to be inefficient, and plagued by usability problems. The dominating communication that takes place through faxing, digital or not, is between municipal home care and the regional health care system. Thus, the nurses’ intentions and attitudes on using the fax are not voluntary but driven out of necessity in their day-to-day work; it was simply the only way for them to do the job. The home care nurses experience a dependency status towards the regional health care system, which often functions to their disadvantage [24]. The technology has been replaced but the practice itself remains mostly the same as before the pandemic (i.e., the performance impact has not improved) [27]. The use of digital faxing is thus mandatory and driven top-down (i.e., not by the nurses themselves). Future studies should delve deeper into how faxing affects the working conditions of home care nurses.

TAM postulates that users have agency in their use of technology (i.e., a choice to use the system or not). However, as described above, this is not necessarily always the case. In the public sector, it is not uncommon for professionals to be forced to use technology even though its use is not beneficial for them. Under these conditions, TAM does not fully explain why technologies become used. Here TTF better explains the effects that technology has on work. “Utilization” and “performance impact” in TTF may be high for digital faxing. However, when in combination with their usability problems, this shows that these parameters, by themselves, are not necessarily good predictors of good working conditions.

In contrast to digital faxing, the use of digital meetings is driven more by the nurses themselves and on their terms (i.e., bottom-up). Given the right conditions, the nurses strive for work efficiency which provides opportunities for flexibility and education (Table 3). Rather than replacing physical meetings, the digital meetings continue to coexist as a middle ground between physical meetings and phone calls. However, among the nurses there is a vigilance concerning what the adoption of digital meetings could mean for their future practice when interacting with other professional groups, such as licenced practical nurses and nursing assistants in homemaker services, and general practitioners at community health centres. On the one hand, it is acknowledged that the benefit of formalizing communication with homemaker services, and for time savings, can be at the cost of the social relationships the nurses have with them. On the other hand, towards community health centres, there is the risk of losing the small amount of physical contact the nurses have together with general practitioners.

The implementation processes of the new technologies were initially solely motivated by the emergency brought on by the pandemic. The municipalities had no choice but to embrace the new technological solutions to “survive”. In the “new normal” world, the existing driver behind the implementation process of the past was no more, and the adoption of a new process proceeded. Two adoption processes have in fact taken place where the properties of technology, “technology characteristics” according to TTF, has been constant. As the COVID-19 imbued conditions changed over time, this brought changes to the “task characteristics” which in turn affected the technologies context of use and the “task-technology-fit”. If the technologies could find a place in the new practice, they were kept. If not, they were abandoned.

Among the three new technologies encountered in this study, digital faxing was the one that had the most impact on the nurses’ day-to-day work (see e.g. Table 2). Once implemented, the municipalities could not reverse the process and go back to physical faxing machines when the practice returned to normal. As before, there was only one main option for the nurses to communicate with the regional health care system, and that was the fax.

What is interesting here, is that digital faxing did not seem to provide any additional usability-related gains. Rather, its use in practice was very similar to the process applied when using the previous analogue fax. The term “usability”, of which utility is a central aspect, is related to “perceived usefulness” in TAM. Usability is defined as “the extent to which a system, product or service can be used by specified users to achieve specified goals with effectiveness, efficiency and satisfaction in a specified context of use” [47]. From this perspective, in situations where technology has good utility, the more inclined users become to use it. In addition, as a technology becomes more impactful on performance the more utilized it becomes, and the better it fits the tasks. However, as mentioned before, this does not necessarily mean that it contributes positively to the work environment. Neither is “performance impact” isolated to “the accomplishment of a portfolio of tasks by an individual” [27]. For workers with a dependency status, as the home care nurses have with the regional health care system, the continued use and impact of the fax, e.g., is a result of a shortage of communication alternatives. Where usability problems exist, the use of the technology is still necessary, thus creating a forced use onto the nurses [24].

The other main factor in TAM – “perceived ease of use” – has much in common with “user experience”. User experience (UX) is defined as “[the] user’s perceptions and responses that result from the use and/or anticipated use of a system, product or service” including “…emotions, beliefs, preferences, perceptions, comfort, behaviors, and accomplishments… [occurring] before, during and after use.” [47]. In other words, “perceived ease of use” relates to user satisfaction, which can influence the intents behind use among new users of technology [48]. As users become more familiar, “perceived usefulness” takes over in driving the technology’s continued use. The overlap between technology acceptance and UX is important to research because emotions and experiences interact with the utilization of technology use. This is becoming more recognized as important to performance and wellbeing [49].

The reasoning behind the progressive discovery of the new technologies’ utility in the “new normal” is not only related to the experience nurses obtained using the technologies themselves. It is also related to their knowledge of how similar interactions have worked contextually and affected their work situation in the past [23–24]. This introduces expectations, good and bad, on how new technology will be adopted, and interactions enacted through them. Cognitively, this can be explained with Neisser’s Perception Cycle Model (PCM) [50]. According to PCM, peoples’ actions and decisions are shaped by their internal mental templates (schemata) and the information in the surrounding environment (world). In other words, people have preconceptions, experiences, and agendas in the context that has existed before the implementation of any new system. When individuals are introduced to a new technology for interaction, its adoption and use is partly affected by how those interactions have previously worked. Hence, context is key. However, it is interesting that information about the users, systems, tasks, and organizational context is often missing in the literature despite its wide recognition of being important [49, 51]. To some extent, this can be argued similar to the “experience” factor introduced in the extensions of TAM, namely TAM2 and TAM3 [32–33]. However, “experience” in TAM2 and TAM3 is more related to the experience with the actual system and has no feedback loop from descendent factors in the models. In other words, there are indications that “attitudes towards use”, “behavioural intentions” and “actual system use” in TAM and “performance impact” in TTF can influence antecedent factors in the respective models’ based on how technologies are used and affect work in practice.

### Strengths and limitations

One of the strengths of this study is that it investigates technology acceptance and use over time, an approach seldom found in previous studies. This provides a unique opportunity to follow how work and related utilization of technology dynamically changed due to the conditions brought on by the COVID-19 pandemic. This study has been conducted in a care setting where society’s most vulnerable groups are being cared for – and where many died during the pandemic. At the same time, home care is a healthcare setting that has not been extensively researched. Thus, a second strength is that this study provides well-needed knowledge on work in a care setting that it not well understood.

The theoretical framework used to interpret the research findings have encompassed the TAM and TTF models. Employing the more comprehensive UTAUT model could have given additional knowledge about how social- and environmental factors can influence technology adoption, including understanding of possible challenges and technology enablers [34]. However, this would require a much more extensive set of data probably including quantitative survey data to apply the model successfully. Thus, we do not find it suitable as a framework for this type of qualitative study.

Generalizability of qualitative and ethnographic research has on occasion been criticized [52]. This study presents the situation and technology adoption in four of Sweden’s 290 municipalities during the COVID-19 pandemic. The authors do not argue the findings to automatically be representable of the situation in all Swedish home care organizations. However, analytic generalizability is possible by relating the research findings to established theoretical models [53]. Furthermore, transferability, i.e. case-to-case transfer, is made possible by providing descriptions that are thick and rich in detail [53]. The research findings are likely applicable to settings where the reader recognizes their own situation in the given descriptions.

## Conclusions

The aim of this article was to investigate how new digital technology was accepted and used by Swedish registered- and community health nurses in municipal home care during the COVID-19 pandemic. The results show that the technology had a central role for the provision of home care during the pandemic and that the contextual conditions under which technology was used were not static, but dynamic and changing over time. Experience and conditions in the environment had a major impact on technology acceptance and use. As conditions in the environment changed, so did the motivation to use the technology. This is not captured well in the TAM and TTF theoretical frameworks that describe more linear relationships. Since antecedent factors in implementation processes are dynamic, not static, and feedback loops (i.e., experience) appear to play an important role, these factors need to be addressed in future technology acceptance models. Additionally, as the results show, users do not necessarily have agency in relation to their use of technology but are rather mandated to do so. Coupled with bad usability and a strained work environment, this could potentially affect the employees negatively. Thus, a more holistic discussion is needed where context, feedback, agency, and control at work are given more consideration and space.

## Data Availability

The data set used and analysed for the study is not publicly available due to the privacy and confidentiality of the participants information, but it is available from the corresponding author on reasonable request.
